# Circadian Reinforcement Therapy in Combination With Electronic Self-Monitoring to Facilitate a Safe Postdischarge Period for Patients With Major Depression: Randomized Controlled Trial

**DOI:** 10.2196/50072

**Published:** 2023-11-27

**Authors:** Anne Sofie Aggestrup, Signe Dunker Svendsen, Anne Præstegaard, Philip Løventoft, Lasse Nørregaard, Ulla Knorr, Henrik Dam, Erik Frøkjær, Konstantin Danilenko, Ida Hageman, Maria Faurholt-Jepsen, Lars Vedel Kessing, Klaus Martiny

**Affiliations:** 1 Mental Health Centre Copenhagen Copenhagen University Hospital Frederiksberg Denmark; 2 Mental Health Centre Copenhagen Copenhagen University Hospital Copenhagen Denmark; 3 Department of Computer Science University of Copenhagen Copenhagen Denmark; 4 Institute of Neurosciences and Medicine Novosibirsk Russian Federation; 5 Mental Health Services Copenhagen University Hospital Copenhagen Denmark

**Keywords:** major depression, internet interventions, self-monitoring, sleep, circadian, chronobiology, chronotherapy, clinician assisted

## Abstract

**Background:**

Patients with major depression exhibit circadian disturbance of sleep and mood, and when they are discharged from inpatient wards, this disturbance poses a risk of relapse. We developed a circadian reinforcement therapy (CRT) intervention to facilitate the transition from the inpatient ward to the home for these patients. CRT focuses on increasing the zeitgeber strength for the circadian clock through social contact, physical activity, diet, daylight exposure, and sleep timing.

**Objective:**

In this study, we aimed to prevent the worsening of depression after discharge by using CRT, supported by an electronic self-monitoring system, to advance and stabilize sleep and improve mood. The primary outcome, which was assessed by a blinded rater, was the change in the Hamilton Depression Rating Scale scores from baseline to the end point.

**Methods:**

Participants were contacted while in the inpatient ward and randomized 1:1 to the CRT or the treatment-as-usual (TAU) group. For 4 weeks, participants in both groups electronically self-monitored their daily mood, physical activity, sleep, and medication using the Monsenso Daybuilder (MDB) system. The MDB allowed investigators and participants to simultaneously view a graphical display of registrations. An investigator phoned all participants weekly to coinspect data entry. In the CRT group, participants were additionally phoned between the scheduled calls if specific predefined trigger points for mood and sleep were observed during the daily inspection. Participants in the CRT group were provided with specialized CRT psychoeducation sessions immediately after inclusion, focusing on increasing the zeitgeber input to the circadian system; a PowerPoint presentation was presented; paper-based informative materials and leaflets were reviewed with the participants; and the CRT principles were used during all telephone consultations. In the TAU group, phone calls focused on data entry in the MDB system. When discharged, all patients were treated at a specialized affective disorders service.

**Results:**

Overall, 103 participants were included. Participants in the CRT group had a significantly larger reduction in Hamilton Depression Scale score (*P*=.04) than those in the TAU group. The self-monitored MDB data showed significantly improved evening mood (*P*=.02) and sleep quality (*P*=.04), earlier sleep onset (*P*=.009), and longer sleep duration (*P*=.005) in the CRT group than in the TAU group. The day-to-day variability of the daily and evening mood, sleep offset, sleep onset, and sleep quality were significantly lower in the CRT group (all *P*<.001) than in the TAU group. The user evaluation was positive for the CRT method and the MDB system.

**Conclusions:**

We found significantly lower depression levels and improved sleep quality in the CRT group than in the TAU group. We also found significantly lower day-to-day variability in daily sleep, mood parameters, and activity parameters in the CRT group than in the TAU group. The delivery of the CRT intervention should be further refined and tested.

**Trial Registration:**

ClinicalTrials.gov NCT02679768; https://clinicaltrials.gov/study/NCT02679768

**International Registered Report Identifier (IRRID):**

RR2-10.1186/s12888-019-2101-z

## Introduction

Patients with major depression exhibit various circadian rhythmic abnormalities. These include day-to-day instability, drifting of the sleep-wake cycle [1-3], diurnal change in mood [4], alteration in cortisol rhythm [5], and changes in the circadian pattern of gene expression and metabolism in the brain [6,7]. Furthermore, mood symptoms are closely linked to the timing of sleep, and sleep delay often causes mood deterioration and sleep phase advancement, generating an antidepressant effect [1,8,9]. The circadian system depends on the external zeitgebers (time cues) to maintain rhythmicity and entrainment with the external light-dark cycle. A low zeitgeber input can cause drifting of sleep and irregularities in the sleep-wake cycle and mood [1,10,11], and adequate zeitgeber input can reset and stabilize rhythms [12]. These mechanisms are described in detail in the published protocol of this study [13].

For patients with a major depressive episode, the transition from an inpatient psychiatric ward to an outpatient status poses a risk of relapse, readmission, suicide attempts, and suicide [1,14,15]. Therefore, various outpatient mental health services that receive patients shortly after discharge have been developed to manage this challenge [16].

To deliver timely help, researchers have made attempts to monitor patients’ daily condition throughout this transition to catch signs of deterioration and provide immediate intervention. We previously tested an electronic system (Daybuilder [17]) using daily entering of mood, sleep, and activity data in a one-arm feasibility trial [1] in patients with a major depressive episode. In that study, participants logged data daily, covering a 4-week transition phase from inpatient to outpatient status. The logged data automatically generated a graphic display of all the variables to help participants understand the temporal patterns of the data. Clinicians telephoned the participants weekly to discuss compliance with the data entry and patterns in the graphical display. The results showed a readmission rate of 13% and that the sleep midpoint drifted 39 (SE 10) minutes later over the 4-week period (*P*<.001), and this drift was associated with a significant worsening of mood (*P*=.03). Another evident result of that study was that both mood and sleep were very unstable on a day-to-day basis. The usability of Daybuilder was very high, with a System Usability Score (SUS) of 86.2 (SD 9.7).

A newer randomized controlled trial in patients with major depression used the Monsenso system, a smartphone-based electronic monitoring system [17]. Using this system, participants and clinicians could monitor their daily mood and sleep after discharge. No effect on readmission was found, but participants in the active group experienced better recovery than those in the control group, as measured by the Recovery Assessment Scale [15].

It is debatable whether electronic monitoring per se has a positive or negative impact on patients with affective disorders [18,19]. For example, in patients with bipolar disorder, monitoring of negative mood might sustain the level of depressive symptoms; on the other hand, continuous symptom monitoring might also enable patients to acknowledge their condition in due time and receive timely help [15,20,21].

Relevant literature showed that technology-based self-help and minimal contact therapies have been proposed as effective and low-cost interventions for anxiety and mood disorders [22], and the Daybuilder electronic system is created with clinician support facilities built into the system.

This study aimed to combine a newly developed intervention, Circadian Reinforcement Therapy (CRT) [13], with data logging in an electronic system and clinician feedback. CRT intervention is described in detail in the published protocol of this study [13]. CRT strengthens zeitgeber inputs to the circadian system through social contact, physical activity, diet, and daylight exposure and provides sleep guidance, aiming to stabilize mood and prevent drifting of sleep timing. The intervention was delivered through specialized psychoeducation supported by an electronic monitoring system. This is the first study to test CRT intervention.

The electronic system used in this study is a slightly updated version of the Daybuilder electronic system used in our previous study [1]. The updated Daybuilder system was acquired by Monsenso in 2014 [17] and is therefore named Monsenso Daybuilder (MDB). There are only minor differences between the Daybuilder and MDB systems, including a graphical visualization of day-to-day variability and a more detailed description of depression parameters.

This study aimed to prevent the worsening of depression in patients with a major depressive episode after discharge from inpatient psychiatric wards [13]. We hypothesized that the CRT intervention would help patients recover faster and would reduce the likelihood of relapse in the period after discharge. We did not expect the electronic system to act as a treatment modality, but rather as a medium to facilitate the new CRT intervention.

## Methods

### Study Design

This study was designed as a 4-week, randomized, controlled, single-blinded, parallel-group trial, with a balanced allocation rate (1:1) to either the CRT or the treatment-as-usual (TAU) group.

The procedure of this study was described in a published study protocol [13]. We developed a CRT based on the knowledge from chronobiology and chronotherapy of zeitgebers, light pathways in the brain and their relationship to mood [23], the phase response curve of light [12], and sleep regulation [24]. There is evidence that not only are rhythms such as sleep and melatonin and temperature curves distorted in major depression, but there are also changes in circadian gene expression in the brain [7]. The elements in CRT can be considered as an extension of the social metrics therapy developed by Frank et al [25] but with a focus on zeitgeber inputs. Thus, CRT focuses on the extent and timing of the inputs to the circadian system. Associated psychoeducation and written information aim to help patients understand the importance of the circadian system, including the need for zeitgeber inputs to sustain sleep and mood. The purpose of CRT is to stabilize sleep and mood, prevent sleep drift, and induce a slight sleep phase advance to help induce an antidepressant response [26-28].

### Participants

Participants who aged ≥18 years and had a diagnosis of a major depressive episode, according to the Diagnostic and Statistical Manual of Mental Disorders, Fourth Edition [29], were eligible for inclusion after signing informed consent. Exclusion criteria were a score of ≥2 on the Hamilton suicidal item 3, current abuse of alcohol or illegal substances, comorbid dementia or other brain disorders that would make the use of the MDB difficult, bipolar disorder, and psychotic depression.

**Figure 1:**
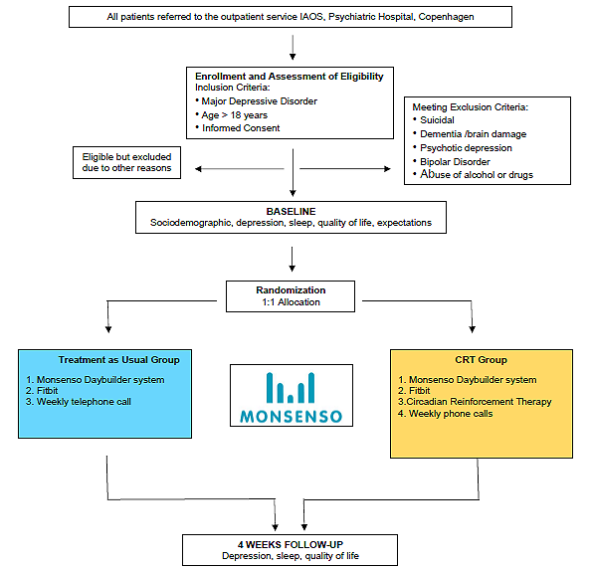
Flowdiagram. IAOS: Intensive Affective Outpatient Service.

All the patients who were referred from the psychiatric inpatient wards at the Mental Health Center Copenhagen for follow-up at the Intensive Affective Outpatient Service (IAOS) were consecutively asked to participate in the study. The IAOS exclusively receives patients with depression immediately after discharge from inpatient psychiatric wards (Figure 1). We aimed to include the patients when they were still in the inpatient ward or at the latest 3 weeks after commencing treatment at the IAOS.

### Interventions

Using a computer-generated random list without stratification, we randomized the patients to either the group using the CRT intervention as an add-on to the ongoing IAOS treatment or the group receiving only the IAOS treatment (TAU). Treatment at the IAOS included individual consultations with psychiatrists, supporting sessions with specially trained psychiatric nurses, group psychoeducation including some sleep hygiene advice (but no information on circadian-related components), physiotherapy, and pharmacological treatment.

All participants in both the groups used the MDB self-monitoring system to assess daily mood, sleep, activity, and medication compliance [1,30]. In addition, all participants were given a wearable Fitbit activity tracker (model charge 2) to measure the number of steps and heart rate.

### CRT Group

**Figure 2:**
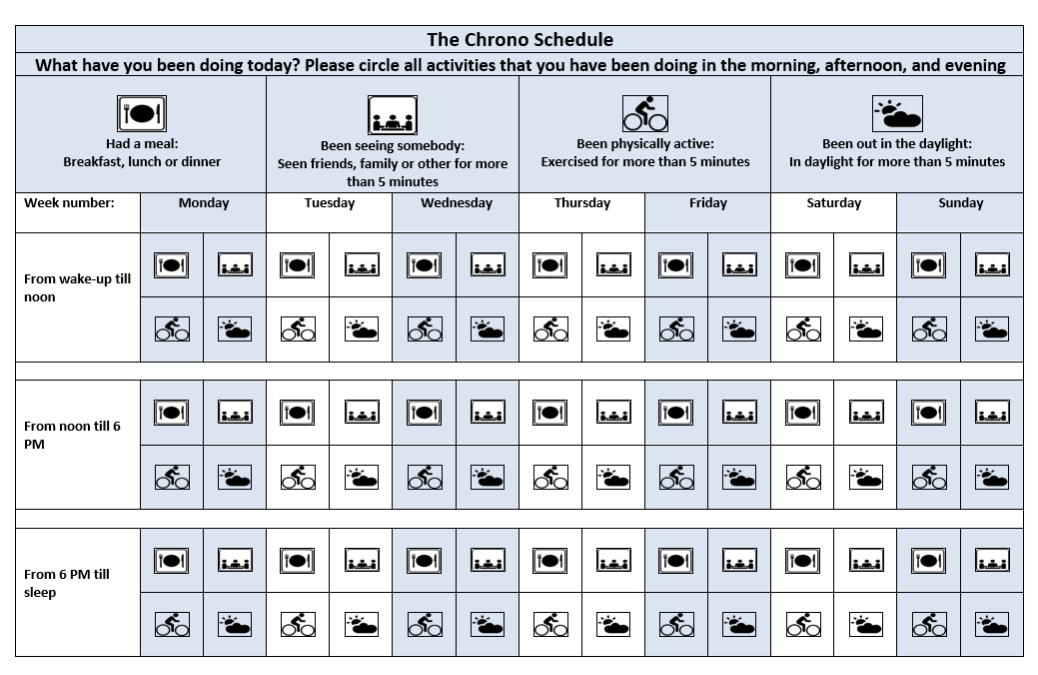
Chrono Schedule.

Participants allocated to the CRT group were given specialized psychoeducational sessions at the beginning of the trial period to facilitate the implementation of the CRT principles. These sessions were often given for 2 days to facilitate learning, with booster sessions at weekly phone calls. The sessions were scheduled as a 1-hour individual presentation, based on PowerPoint (Microsoft Corp) slides, where the principles of CRT were explained. The presentation was supplemented with paper-based informative material and leaflets. This specialized psychoeducation was intended to provide participants with knowledge on ways to strengthen time signals to their circadian system using temporally structured and higher levels of exposure to zeitgeber elements, such as daylight, exercise, regular meals, and social contact [11,31-33]. CRT psychoeducation also includes sleep hygiene guidance, such as advice on trying to avoid naps that is often associated with deterioration of mood, a phenomenon named “nap-mood-drop” [34]. If napping could not be avoided, the advice was to nap <30 minutes to help increase the sleep pressure. Advice was also given regarding earlier sleep timing to induce an antidepressant effect. In addition, a paper *chrono diary* (Figure 2) was used to help the participants follow their own implementation of the instructed zeitgeber elements. The participants were directed to check the boxes in the chrono diary daily, indicating when they had engaged in various activities, such as consuming a meal, spending time with someone for >5 minutes, engaging in physical activity for >5 minutes, and being in sunlight for >5 minutes. This was performed at 3 distinct intervals during the day: from the time of awakening until noon, from noon until 6 PM, and from 6 PM until sleep onset [13]. Data from the chrono diary were not entered into the MDB system but were used in weekly phone calls. An individualized plan for future weeks was made for each participant in the CRT group, focusing on 3 self-selected elements (eg, awakening at 7 AM, having exposure to daylight, and exercising or walking). An action plan covering how to handle deterioration was also developed.

Once a week, an investigator phoned or met participants at the IAOS, and together, they examined the self-assessment data from the last week through the graphical display in the MDB system and discussed possible solutions using the elements in the CRT method. In addition, MDB data entries from participants in the CRT group were monitored by the investigators on all weekdays, and predetermined triggers elicited additional phone calls to participants. The trigger points were sudden mood drops, changes in sleep timing, reduced medication compliance, low data entry compliance, and negative text messages (Multimedia Appendix 1). CRT principles are described in more detail in the protocol [13], and the usability of the MDB system is described by Lauritsen et al [1].

CRT methods were used in all phone calls in the CRT group to help stabilize mood, sleep, and activity by recalling CRT elements that could strengthen zeitgeber signals. For example, an observed drift to late sleep onset would elicit advice on morning daylight exposure, morning exercise, avoiding late-night light exposure, late-night exercise, and late meals. Likewise, unstable sleep patterns would elicit advice on more regular sleep schedules and examine the underlying reasons for deviations (being out late at night, eating late, and ruminating when going to bed).

During the planned weekly phone calls, the investigator also evaluated and discussed (1) experience with the MDB system, (2) suicidal ideation, (3) side effects of medications, (4) worsening of depression and plan for action, and (5) compliance with data entry.

### TAU Group

Patients in the TAU group also received weekly telephone calls, and these talks only covered the items (1) to (5) that are mentioned in the previous paragraph for the CRT group. See the published study protocol for more details [13].

### Outcomes

#### Questionnaires and Self-Monitoring

The Danish version of the Mini International Neuropsychiatric Interview for Diagnostic and Statistical Manual of Mental Disorders, Fourth Edition was used for diagnosis [35]. The investigators were certified using the Mini International Neuropsychiatric Interview. Sociodemographic data were collected at the baseline. All participants were interviewed using the Hamilton Depression Rating Scale (HAM-D_17_) [36] by a blinded investigator at baseline and end points. The following self-assessment scales were performed by the patient at baseline and the end point: Major Depression Inventory (MDI) [37] (score range 0-50, 50=severest depression), the World Health Organization Well-Being Index (WHO-5) [38] (score range 0-100, 100=best quality of life), and the Morningness-Eveningness Questionnaire (MEQ) [39] that assesses preferred time points for defined tasks as a measure of chronotype (with scores <42 indicating *evening type*, 42-58 indicating *intermediate type*, and >58 indicating *morning type*). The Pittsburgh Sleep Quality Index (PSQI) assesses sleep quality during the last month (a global score of ≥5 signifies poor sleep quality) [40]. The SUS scale (10 questions rated based on agreement with statements from 1 to 5 and converted to a score range from 0 to 100; 100=best) [1,41] was used at the end point to evaluate the MDB system. Participants in both the groups logged self-assessment data into the MDB system daily for a 4-week period. The procedure has been described in more detail in the protocol [13]. The following parameters were logged in the MDB system: depression levels assessed at the time of awakening “depression_morning,” depression levels assessed just before bedtime “depression_evening,” and depression levels assessed as a mean for the last day and entered before bedtime “depression_daily,” activity as the number of steps in the last 24 hours from the Fitbit bracelet, daily medication adherence with categories of yes or no, sleep onset, sleep offset, number of awakenings, sleep quality, and daytime naps with time points for nap start and end. Mood and sleep quality were monitored using a scale, with response categories ranging from 0 to 10, where 10 indicates the best quality. Text comments, viewable to participants and investigators, could be made next to the data entries, for example, as additional information on sleep or mood problems. In the CRT group, a text comment option “sleep-wake cycle” was specifically designed so that the participants could describe what measures they used each day to stabilize the sleep-wake cycle. This was used on each phone call to evaluate the participants’ use of CRT principles in the previous week.

The wearable Fitbit bracelet is a wrist-worn data logger with a display that counts the number of steps. The number of steps was entered into the MDB system each night by the participant, covering the last 24 hours. Pulse data from the Fitbit bracelet were read through a computer interface at the end of the visit.

At baseline, we administered an Expectation of Treatment Outcome scale, with a score range from 0-10, where 0 signifies unchanged levels of depression and 10 no depression). In addition, a short questionnaire concerning diurnal patterns of depression symptoms was administered. At the end point, all participants were asked questions to evaluate their general experience in the study period, and participants in the CRT group were also asked CRT-specific questions.

#### Biochemical Outcomes

A subset of participants performed repeated evening saliva sampling to assess the Dim Light Melatonin Onset (DLMO) at baseline and end point. Oral and written instructions on how to perform saliva sampling were provided. This included securing dim light exposure during the sampling period and avoiding tea, caffeine, cola, bananas, chocolate, smoking, and fluids with colorants from 1 PM to the end of the sampling period. In addition, participants were advised not to brush their teeth during the sampling period and not to use drugs containing aspirin or ibuprofen for the entire sampling day. Saliva samples were taken at home beginning 5 hours before expected bedtime and thereafter every half hour, with the last sample collected 1 hour after expected bedtime, summing up to 13 samples. Instructions were provided by KD. Samples were frozen at −20 °C until analysis. The sensitivity of melatonin assay is highly variable. The IBL RE54041 enzyme-linked immunosorbent assay used in this study was considered valid [42].

DLMO was determined using the hockey-stick method [43]. The phase angle difference (PAD) [44] was calculated as the difference between the mean of the last 3 days of midpoint sleep and DLMO for each participant. The sleep midpoint was calculated from the entered sleep onset and offset data in the MDB system.

#### Ranking of Outcomes

The primary outcome was defined as the difference between the 2 groups in terms of changes in HAM-D_17_ scores from baseline to end point. The secondary outcome was defined as the difference between the 2 groups in terms of changes in the MDI scores from baseline to end point. The tertiary outcome was defined as the difference in the PAD magnitude between the groups at the end point. Explorative outcomes were defined as the difference between the estimated end point scores of the self-monitored data from the MDB system, activity data from the Fitbit, and the WHO-5 scale scores of the CRT group and those of the TAU group. The MEQ and PSQI scores were used as descriptive data and covariates in the models.

### Statistics

The intention-to-treat method was applied using all data for all included participants in all analyses. Interviewer-based and self-reported paper-and-pencil data with baseline and end point assessments were analyzed using a general linear model (GLM), with end point scores as the outcome. Data from the MDB system were analyzed using a mixed model for repeated measures, using all available data without imputation. In the GLM and mixed model, covariates were removed by backward selection, depending on the magnitude of the parameter estimate and significance. The level of significance was set at a *P* value of .05. Continuous baseline characteristics were compared using a 2-tailed *t* test or Wilcoxon 2-sample test, depending on the data distribution. Categorical data were analyzed using the chi-square test.

For analyses of day-to-day variability of sleep data, the data were truncated, excluding periods of sleep onset before 6 PM and sleep offset after 6 PM.

In the GLM model and the mixed model for repeated measures, the following covariates were used: baseline values of the outcome, the treatment group, interaction term between day and the 2 treatment groups, time from discharge to visit 1 (TDV1), interaction term between TDV1 and the treatment groups, electroconvulsive treatment (ECT; yes or no), interaction term between ECT and the treatment groups, medication adherence (yes or no), and MEQ scores.

The covariates were chosen based on the assumption of the influence on the respective outcomes in the models: ECT was selected because the resultant cognitive disturbances might make CRT psychoeducation less effective [45] and TDV1 as it was the primary assumption in the study that CRT should be implemented as a preventive measure as early as possible after discharge to influence the depression course. Medication compliance was included because of its possible effect on depression levels [46], while MEQ was included, as this is assumed to be linked to sleep timing and thus mood [47].

Day-to-day variability of the MDB data was estimated as residuals (covariance parameter estimates) for each group in a repeated-measures model. Statistical significance was calculated from the difference in log-likelihood values in a random versus repeated model as a chi-square value with 1 degree of freedom.

### Ethical Considerations

The study was approved by the Regional Committee on Health Research Ethics (H-15013943) and Danish Data Protection Agency (2012-58-0004/RHP-2016-015 I-suite number 04734). All participants provided written informed consent before enrollment in the study. Care was taken to evaluate whether the participants found the study procedures stressful. Daily self-monitoring can cause stress and inconvenience, and participants could withdraw from the study at any time.

## Results

### Sociodemographic and Baseline

A total of 342 patients were screened for participation, and 103 gave informed consent to participate in the study from September 2016 to November 2020, with 51 participants randomized to the CRT group and 52 participants in the TAU group. One participant withdrew informed consent before enrollment, and one participant’s inclusion was canceled because of the decision to extend the inpatient stay. Both the participants were randomized into the CRT group. No outcome data were collected for these 2 participants. Discharge date was missing for 3 participants. Overall, 29% (29/98) of the participants were included on or before the day of discharge, 56% (55/98) on or before day 8 after discharge, 60% (59/98) on or before day 15 after discharge, 71% (70/98) on or before day 22 after discharge, and 78% (76/98) on or before day 29 after discharge. Sociodemographic data in Table 1 show that the parameters were evenly distributed between the 2 groups except for the Expectation of Treatment Outcome scale score that was significantly higher in the CRT group than in the TAU group (CRT: mean 5.9, SD 2.5; TAU: mean 4.7, SD 2.5; *P*=.03).

Of the remaining 101 participants, 15 (14.9%) did not attend the end point assessment visit (9 participants in the CRT group and 6 in the TAU group) and could therefore not be included in the analyses based only on the questionnaires administered at baseline and end point visits. However, their MDB data were used for exploratory analyses. The reasons for missing the end point assessment in the CRT group were given as “feeling participation too stressful or burdensome” (n=4) and “deterioration of depression with follow-up not possible” (n=4), and 1 participant was lost to follow-up; and the reasons in the TAU group include “deterioration of depression with follow-up not possible” (n=5) and “end point assessment delayed for practical reasons” (4 months; n=1).

A total of 8 (8%) participants were readmitted to the inpatient psychiatric ward during the study period, with 3 (6%) in the CRT group and 5 (10%) in the TAU group. These participants continued their study procedures when possible and were assessed at the end point. Two participants, both from the TAU group, took a nonfatal overdose due to suicidal ideation (one took venlafaxine plus an antihypertensive drug and one oxazepam). One of these participants dropped out of the study, and the other completed the study. Both the incidents were reported to the regulatory authorities. Score data from 7 Hamilton interviews were lost when they were sent between the 2 departments. Thus, for the analysis of the primary HAM-D_17_ outcome, data from 79 participants were available, of whom 35 were allocated to the CRT group and 44 to the TAU group. For the analyses of the secondary outcome on the MDI scale, data from 81 participants were available for analyses, of whom 39 were in the CRT group and 42 in the TAU group. For the explorative self-monitored data in the MDB system, 45 participants in the CRT group and 50 in the TAU group logged the data for one or more days. Compliance with the data logged into the MDB system was 90.7% (2565/2828) for the depression_daily measure (of the 101 eligible participants), 82.49% (2333/2828) for activity data, and 97.81% (2766/2828) for sleep onset and offset. The questionnaire examining the diurnal patterns of depression symptoms showed that 89.8% (44/49) in the CRT group and 73.1% (38/52) in the TAU group experienced a characteristic change in depression severity during the day. “Best-in-the-morning” pattern was experienced in 14.3% (6/42) and 21.2% (7/33), “best-at-noon” in 31% (13/42) and 30.3% (10/33), and “best-at-evening” in 54.8% (23/42) and 48.5% (16/33) in the CRT and TAU groups, respectively (between groups, *P*=.72).

**Table 1:** Sociodemographics.

Parameter		CRT^a^ group (n=50)	TAU^b^ group (n=52)
Age (years), mean (SD; range)		41.8 (14.8; 18-70)	40.6 (14.8; 18-74)
Gender (women), n (%)		31 (62)	27 (51)
**Duration of current episode (month)**			
	Values, mean (SD)	11.3 (15.7)	15.1 (19.9)
	Values, median (IQR; range)	6.0 (4.0-11.0; 2-78)	7.0 (3.0-16.5; 1-89)
Recurrent episodes, n (%)		26 (52)	30 (58)
**Number of previous episodes**			
	Values, mean (SD)	2.6 (5.1)	1.5 (1.8)
	Values, median (IQR)	1.0 (0-3.0)	1.0 (0-2.5)
Suicide attempt in connection with actual admittance to inpatient ward, n (%)		10 (20)	10 (19)
One or more previous suicide attempt, n (%)		15 (30)	11 (21)
Patients experiencing an eliciting factor for actual episode, n (%)		45 (90)	47 (90)
Smoking, n (%)		16 (32)	21 (40)
Number of cigarettes per day, mean (SD)		12.1 (6.2)	14.2 (11.3)
ECT^c^ in current episode, n (%)		17 (34)	10 (19)
Number of ECT treatments in current episode, mean (SD; range)		14.6 (6.5; 4-26)	11.8 (3.2; 8-17)
Drinking alcohol, n (%)		29 (58)	28 (54)
Alcohol intake more than once per week, n (%)		6 (12)	10 (19)
Expectation of Treatment Outcome (0-10, with 0=unchanged and 10=no depression), mean (SD)		5.9 (2.5)^d^	4.7 (2.5)

^a^CRT: circadian reinforcement therapy.

^b^TAU: treatment as usual.

^c^ECT: electroconvulsive treatment.

^d^*P*=.03.

### Analysis of the Primary Outcome

The mean HAM-D_17_ scores at inclusion were 15.8 (SE 0.9) and 15.5 (SE 0.8) for the CRT and TAU groups, respectively, ranging from 2 to 31. Analysis of the HAM-D_17_ end point as the primary outcome with the described covariates (see the *Statistics* section) showed no significant effects of ECT or MEQ scores, and these covariates were removed. The final model showed a significantly larger reduction in HAM-D_17_ scores in the CRT group than in the TAU group (*F_1_*-score=4.2; parameter estimate=2.6; *P*=.04). Furthermore, there was a statistically significant interaction between TDV1 and the groups (*F_1_*-score=8.5; parameter estimate=−0.08; *P*=.005). For participants included at discharge (TDV1=0), the parameter output thus showed an estimated difference in end point HAM-D_17_ scores of 2.6 points, with a gradually diminishing effect of −0.08 HAM-D_17_ points per day. This shows that earlier implementation of CRT intervention was associated with a greater antidepressant end point difference between the groups. The estimated end point HAM-D_17_ values for the CRT and TAU groups were 12.8 (SE 0.7) and 13.6 (SE 0.6; Table 2), respectively. The HAM-D_17_ baseline used in the estimation model is the whole sample mean 15.6 (SE 0.6).

The model fit for the primary outcome showed 2 observations with a high Cook distance, suggesting influential data (participants 35 and 45). Sensitivity analyses with the removal of these 2 observations showed a continued statistically significant effect (*P*=.04), and the observations were included in the analysis. The baseline HAM-D_17_ score for the 15 participants who did not attend the end point assessment was 16.8 (SD 6.5) compared with 15.4 (SD 6.0) for those who did.

**Table 2:** Results from interviewer and self-assessment scales.

Instrument and visit			CRT^a^ group	TAU^b^ group
**Outcome measures**				
	**HAM-D_17_^c^ (n=79), mean (SE)**			
		Whole sample baseline	15.6 (0.6)	15.6 (0.6)
		Estimated end point	12.8 (0.7)^d^	13.6 (0.6)
	**MDI^e^ (n=81), mean (SE)**			
		Whole sample baseline	21.5 (1.2)	21.5 (1.2)
		Estimated end point	18.4 (1.1)	20.6 (1.1)
	**WHO-5^f^ (n=81), mean (SE)**			
		Whole sample baseline	40.9 (2.5)	40.9 (2.5)
		Estimated end point	46.3 (2.6)	43.4 (2.2)
**Chronotype and sleep quality**				
	**MEQ^g^ (n=81), mean (SD)**			
		Baseline	49.8 (10.9)	51.2 (11.9)
		End point	52.3 (9.8)	52.1 (11.3)
	**PSQI^h^ (n=84), mean (SD)**			
		Baseline	8.8 (3.6)	10.2 (4.7)
		End point	7.7 (3.0)	9.3 (4.3)

^a^CRT: circadian reinforcement therapy.

^b^TAU: treatment as usual.

^c^HAM-D_17_: Hamilton Depression Rating Scale.

^d^*P*=.04.

^e^MDI: Major Depression Inventory.

^f^WHO-5: World Health Organization Well-Being Index

^g^MEQ: Morningness-Eveningness Questionnaire.

^h^PSQI: Pittsburgh Sleep Quality Index.

### Analyses of the Secondary Outcome

The mean MDI scores at inclusion were 21.7 (SE 1.7) and 21.3 (SE 1.6) for the CRT and TAU groups, respectively. Analyses showed no statistically significant effect of any covariate on MDI scores. The estimated end point MDI values for the CRT and TAU groups were 18.4 (SE 1.1) and 20.6 (SE 1.1; Table 3), respectively. The MDI baseline used in the estimation model is the whole sample mean 21.5 (SE 1.2).

**Table 3:** Results from the self-monitored parameters in the Monsenso DayBuilder system.

Parameter and visit		CRT^a^ group	TAU^b^ group
**Depression_daily (all data), mean (SE)**			
	Whole sample baseline	5.6 (0.2)	5.6 (0.2)
	End point	7.7 (0.4)	7.6 (0.4)
**Depression_morning (all data), mean (SE)**			
	Whole sample baseline	5.6 (0-3)	5.6 (0-3)
	Estimated end point	6.6 (0.4)	6.8 (0.4)
**Depression_evening (all data; medication adherence: yes), mean (SE)**			
	Whole sample baseline	6.1 (0.3)	6.1 (0.3)
	Estimated end point	7.2 (0.2)^c^	6.6 (0.2)
**Depression_evening (all data; medication adherence: no), mean (SE)**			
	Whole sample baseline	6.1 (0.3)	6.1 (0.3)
	Estimated end point	8.3 (0.4)^d^	7.7 (0.4)
**Sleep quality (all data), mean (SE)**			
	Whole sample baseline	6.2 (0.2)	6.2 (0.2)
	Estimated end point	7.0 (0.2)^e^	6.5 (0.2)
**Sleep onset (all data), mean hour:minutes (SE)**			
	Whole sample baseline	23:26 (0:09)	23:26 (0:09)
	Estimated end point	23:17 (0:09)^f^	23:42 (0:09)
**Sleep offset (all data), mean hour:minutes (SE)**			
	Whole sample baseline	7:23 (0:08)	7:23 (0:08)
	Estimated end point	7:50 (0:08)	7:47 (0:08)
**Sleep duration (all data), mean decimal hours (SE)**			
	Whole sample baseline	7.9 (0.1)	7.9 (0.1)
	Estimated end point	8.4 (0.1)^g^	7.9 (0.1)
**Sleep wakeups (all data), numbers (SE)**			
	Whole sample baseline	1.5 (0.2)	1.5 (0.2)
	Estimated end point	2.3 (0.5)	2.5 (0.6)
**Steps from Fitbit (all data), number (SE)**			
	Whole sample baseline	5857 (479)	5857 (479)
	Estimated end point	9195 (764)	9151 (737)

^a^CRT: circadian reinforcement therapy.

^b^TAU: treatment as usual.

^c^*P*=.02.

^d^*P*=.04.

^e^*P*=.04.

^f^*P*<.001.

^g^*P*=.005.

### Analyses of the Tertiary Outcome

Melatonin assessments were performed only in 12 participants (5 in the CRT group and 7 in the TAU group, with 1 missed assessment at the end point in each group) due to COVID-19–related restrictions. For participants with both baseline and end point DLMO assessments, the mean DLMO at baseline and end point was 19:40 (SD 1:10) and 19:27 (SD 0:46) hour:minutes in the CRT group (n=4) and 19:38 (SD=1:06) and 20:01 (SD=0:56) hour:minutes in the TAU group (n=6) (*P*=.30).

For participants with DLMO assessment at both baseline and end points, we analyzed the first 3 days of sleep midpoint during baseline assessment and the last 3 days of sleep midpoint from end point assessment. The analyzes showed a baseline and end point PAD of 7.40 (SD 0.28) and 7.33 (SD 0.53) hours, respectively, in the CRT group and 7.50 (SD 1.20) and 7.03 (SD 1.13) hours, respectively, in the TAU group (*P*=.63).

### Analyses of Explorative Outcomes (Pen-and-Paper Data)

The mean WHO-5 score at inclusion was 40.6 (SE 4.1) and 37.4 (SE 3.3) in the CRT and TAU groups, respectively. We found no statistically significant effect of any covariate on the WHO-5 score. The baseline WHO-5 score used in the estimation model is the whole sample mean of 40.9 (SE 2.5).

The distribution of baseline MEQ chronotypes was 20% (20/98) evening, 57% (56/98) intermediary, and 22% (22/98) morning types for the 2 groups combined. The mean baseline PSQI score was high, indicating poor sleep quality (Table 3).

### Analyses of Explorative Outcomes With Daily Self-Monitored Parameters From the MDB System

Results from the MDB system are shown in Table 3.

Analyses of the depression_daily scores (baseline values, CRT group: 5.7, SE 0.4; TAU group: 5.5, SE 0.4) showed that the only significant covariate was medication adherence (*F_1_*-score*=*5.5; parameter estimate 0.83; *P*=.02), indicating an association between higher depression_daily scores and participants who reported not taking their medication.

For depression_morning depression scores (baseline values, CRT group: 5.5, SE 0.4; TAU group: 5.7, SE 0.3), the only significant covariate was MEQ (*F_1_*-score=7.6; parameter estimate=0.03; *P*=.007), indicating an association between higher (better) depression_morning scores and the morning chronotype.

For the depression_evening scores (baseline values, CRT group: 6.0, SE 0.4; TAU group: 6.3, SE 0.4), we found a significantly higher estimated end point score in the CRT group than in the TAU group (*F_1_*-score=5.7; parameter estimate=0.62; *P*=.02) and a significant effect of medication (*F_1_*-score*=*9.6; parameter estimate 1.1; *P*=.002), indicating a better mood for participants who reported that they did not take their medication.

Analyses of the difference between depression_evening and depression_morning scores showed significantly higher estimated scores (better) in the evening (*F_1_*-score=14.3; parameter estimate=0.36; *P*=.003) as an indication of a diurnal variation.

Analysis of sleep quality showed a significantly larger estimated end point score (baseline values, CRT group: 6.1, SE 0.4; TAU group: 6.3, SE 0.3) in the CRT group than in the TAU group (*F_1_*-score*=*4.6; parameter estimate=0.51; *P*=.04) and a significant effect of MEQ (*F_1_*-score*=*9.9; parameter estimate 0.03; *P*=.002), indicating better sleep quality for patients with a higher MEQ (morning type).

Analysis of sleep onset showed that the estimated end point sleep onset (baseline values, CRT group: 23:28, SE 0:12; TAU group: 23:25, SE 0:12 hours:minutes) was significantly earlier in the CRT group than in the TAU group (*F_1_*-score=7.2; parameter estimate=26.6 min; *P*=.009). The MEQ covariate was significant (*F_1_*-score=21.9; parameter estimate=−0.03; *P*<.001), indicating an association between the morning type and earlier sleep onset.

For sleep offset (baseline values, CRT group: 7:23, SE 0:13; TAU group: 7:23, SE 0:11 hours:minutes), the only significant covariate was MEQ (*F_1_*-score=48.8; parameter estimate=−0.05; *P*<.001), indicating an earlier sleep offset for morning types.

The estimated end point sleep duration (baseline values, CRT group: 7.9, SE 0.18; TAU group: 8.0, SE 0.22) was significantly longer in the CRT group than in the TAU group (*F_1_*-score=8.4; parameter estimate=0.48 [decimal hours]; *P*=.005).

There was no significant difference in the estimated end points between the groups for the number of awakenings (baseline values, CRT group: 1.5, SE 0.2; TAU group: 1.4, SE 0.2).

The estimated mean frequency of naps per day was 15.9% (confidence level [CL] 13.8%-18%) in the CRT group and 21.3% (CL 19.1%-23.4%) in the TAU group (*χ^2^*_1_=12.3; *P*<.001). Estimated mean nap duration was 1.60 (CL 1.0-2.6) hours in the CRT group and 1.77 (CL 1.2-2.7) hours in the TAU group (*P*=.75). The estimated mean timing of naps was 15:08 (CL 14:03-15:13) in the CRT group and 13:18 (CL 12:22-14:13) hours:minutes in the TAU group (*P*=.01).

The baseline values of the steps registered from the Fitbit bracelet were 5221 (SE 630) and 6514 (SE 715) steps in the CRT and TAU groups, respectively. We found no difference in the estimated mean end point number of daily reported steps between the 2 groups, with 9195 (SE 764) and 9151 (SE 737) steps in the CRT and TAU groups, respectively, but showed a significant increase in activity from baseline. In the model, the MEQ was significant (*F_1_*-score=8.2; parameter estimate=97.8; *P*=.005). This indicates a higher level of activity in the morning type (higher MEQ scores).

When comparing the day-to-day variability of MDB data between the CRT and the TAU groups, we found significantly lower values for depression_daily scores with 1.22/1.56 (*P*<.001), no difference between groups for depression_morning scores with 1.42/1.49, significantly lower values for depression_evening scores with 1.45/1.94 (*P*<.001), significantly lower values for sleep onset with 0.85/1.24 (*P*<.001), significantly lower values for sleep offset with 1.03/1.45 (*P*<.001), significantly lower values for sleep quality scores with 1.69/2.15, and significantly lower values for the number of steps with 10,674,014/14,054,624 (*P*<.001).

The mean SUS score was 62.8 (SD 15.2) and 63.3 (SD 16.6) in the CRT and TAU groups, respectively (*P*=.95). The items with the lowest scores were item 4 (“I think that I would need the support of a technical person to be able to use this system”), with a mean score of 3.8 (SD 1.5), and item 7 (“I would imagine that most people would learn to use this system very quickly”), with a score of only 1.9 (SD 1.6).

Triggered extra phone calls were performed in 46% (23/50) of the participants in the CRT group, with a mean number of 1.8 (SD 1.1) calls in the 4-week period per participant. The rules for entries in the MDB that triggered a phone call and the percentage of participants eliciting a specific trigger by MDB entry are shown in Multimedia Appendix 1.

In total, 852 text messages were logged by the participants in the CRT group in the “sleep-wake cycle” text option in the MDB, corresponding to 70.18% (852/1214) of the days. The content of these comments included the CRT elements that the participants had used to strengthen zeitgebers and stabilize the sleep-wake cycle: “went for a walk in the sun,” “doing training,” “visiting friends,” and “keep regular eating hours.” These comments were discussed during the weekly phone calls. The daily compliance with medication was 99.8% (1369/1372) in the CRT group and 96% (1398/1456) in the TAU group. The results of the end point evaluation questions are shown in Multimedia Appendix 2. The text answers were categorized as positive, negative, neutral, or inconclusive. The results showed a significantly higher percentage of positive evaluation to the question, “What has your participation in the project meant for handling your depression after discharge?” in the CRT group than in the TAU group (CRT group: 36/39, 93% vs TAU group: 27/43, 63%; *P*<.001). With regard to the CRT-specific questions, there was a high percentage of positive statements for all questions, with 92% (36/39) recommending the use of the MDB system.

## Discussion

### Principal Findings

We found a significantly larger reduction in the end point Hamilton score, better evening mood, earlier sleep onset, longer sleep duration, better sleep quality, fewer naps, and lower day-to-day variation in sleep and mood in the CRT group than in the TAU group. Furthermore, a statistically significant interaction was found between TDV1 and the groups (CRT and TAU): the longer the TDV1, the lesser the difference between the Hamilton scores in the 2 groups. This supports the intention of this study to include participants as early as possible. Finally, the MEQ scores showed that the morning types had better sleep quality, earlier sleep onset, and sleep offset.

The observed phase advance of sleep in the CRT group compared with the TAU group and the reduced day-to-day variation of sleep were treatment goals and expected through the known impact of zeitgebers on the circadian system, including the sleep-wake cycle. Fewer naps in the CRT group were expected because of psychoeducational advice to limit naps. The significantly earlier timing of naps in the TAU group could be caused by lower sleep quality and shorter sleep duration in this group, resulting in more daytime sleepiness and thus an earlier need for naps.

MEQ associations with sleep timing and quality were also expected, whereas the influence of the morning type on activity is probably a new finding.

Regarding day-to-day variability, our results are in line with our earlier results using sleep time stabilization and bright light, both of which also enhance zeitgeber strength [2].

The intended early inclusion of patients was difficult, with only 56% (55/98) of patients included 8 days after discharge. This delayed inclusion was caused not only by the COVID-19–related restrictions, which made visits to the inpatient wards impossible or difficult but also by the earlier discharge dates than expected in many cases. Thus, in future studies in this field, the implementation of CRT should preferably use a research coordinator located permanently in the inpatient ward to include patients as early as possible and provide psychoeducation sessions before discharge. This might be done in a group session to optimize the use of resources and facilitate group dynamics in learning. If implemented, CRT could also be incorporated into standard psychoeducation in the ward and thus be provided before discharge.

Another finding in the study was that the participants at discharge still had a significant level of depression, as well as cognitive dysfunction, probably making it difficult for them to acquire new information [48]. This was also reflected in the low-to-moderate SUS score, with low scores on the 2 items evaluating learning and support.

The presence of cognitive dysfunction makes it important to refine both psychoeducation and electronic monitoring systems. Compliance with the MDB system was acceptable but might still have caused some frustration.

The structured questions in the end point evaluation showed that the patients felt that the CRT intervention was helpful; thus, the results from the study show that CRT could be used in the management of depression. However, a subset of patients also gave a negative statement regarding the management of the MDB system, and 4 patients dropped out because they felt that it was stressful or burdensome to participate in the CRT group.

Overall, 45% (22/49) of the participants in the CRT group were phoned once or more between weekly scheduled calls due to trigger alerts in the MDB system. This shows that the trigger systems worked, and the evaluation questions regarding phone calls showed that participants appreciated this feature. This might have had a positive influence on the outcomes of the CRT groups and should be included when implementing the system.

Refinement of CRT methods could include video presentations, podcasts, group sessions, peer involvement, and an electronic monitoring system designed as a smartphone app. CRT should be co-designed with users within the framework of a complex intervention [49,50].

Knowledge of how nonphotic zeitgebers influence the circadian system, sleep, and mood work and how they should be administered is still very rudimentary and should be incorporated into CRT refinement. For example, different types of exercise (eg, aerobic or strength training) and their timing and the nutritional components in diet and timing of diet will most likely have different impacts on the circadian system, sleep, and mood [51].

One of the challenges in conducting randomized controlled trials in this patient group is obtaining sufficient, preferably daily, and precise data from the participants without fatiguing them too much. The development of wearable devices is a possible solution for collecting certain data types. We still await an easy-to-use and dependable method to measure sleep and circadian rhythms. The current candidates are ear electroencephalography that are currently being tested [52], 24-hour thermometer measurements [53], and actigraphy with associated light measurement [54].

### Limitations

The sample size was smaller than planned, resulting in less robust findings and limited generalizability. Participants could not be blinded to their allocation to the CRT and the TAU groups, which might have induced an unknown bias in the outcomes. Information on physical comorbidity and types and dosages of medications was not collected, and we cannot rule out an imbalance of these characteristics between groups that could have induced an unknown effect on some of the outcomes. The COVID-19–related restrictions caused difficulty in performing the procedures, and we believe that this might have impaired the implementation of the CRT method. The MDB system might have caused some inconvenience for some patients; nevertheless, their end point evaluation of the system was positive. The 15 patients who did not attend the end point assessment did not deviate substantially in their baseline HAM-D_17_ scores compared with those that were evaluated at the end point, indicating that the sample available for analyses was probably not biased because of these dropouts. We did not have any data on daylight exposure, which would have provided an indication of the signal to the circadian system in the 2 groups, and whether it differed. In future studies, an activity tracker with a light sensor can be used.

### Conclusions

CRT delivered in combination with the MDB system showed a moderate add-on antidepressant effect, the magnitude of which was dependent on the time of inclusion after discharge. Furthermore, we found a sleep phase advancing effect, improvement in sleep quality and evening mood, and a significant reduction in day-to-day variability of mood and sleep. The structured questions from the end point evaluations supported these findings. However, the usability of the MDB system was low for 2 specific items and should be improved. There is thus room for improvement with better electronic systems, timely inclusion, and updated CRT content.

## Appendix

Multimedia Appendix 1 Rules for entries in the Monsenso-Daybuilder system that triggered a phone call to participants in the circadian reinforcement therapy group, with frequency.

Multimedia Appendix 2 User evaluation from semistructured questions at end point.

Multimedia Appendix 3 CONSORT-EHEALTH checklist (V 1.6.1).

## Abbreviations

CL: confidence level
CRT: circadian reinforcement therapy
DLMO: Dim Light Melatonin Onset
GLM: general linear model
IAOS: Intensive Affective Outpatient Service
MDI: Major Depression Inventory
MEQ: Morningness-Eveningness Questionnaire
PAD: phase angle difference
PSQI: Pittsburgh Sleep Quality Index
SUS: System Usability Score
TAU: treatment as usual
TDV1: time from discharge to visit 1
WHO-5: World Health Organization Well-Being Index
