# Supplementary material for: Circadian Reinforcement Therapy in Combination With Electronic Self-Monitoring to Facilitate a Safe Postdischarge Period for Patients With Major Depression: Randomized Controlled Trial
Source: JMIR Ment Health. 2023 Nov 27;10:e50072. doi: 10.2196/50072 (PMC10714270; doi:10.2196/50072)
Supplement: Multimedia Appendix 1 [file mental_v10i1e50072_app1.pdf]

Trigger	Definition	Time frame	Frequency of triggers
**Depression**			
Difference in depression score between morning and evening or between evening and morning	Score drop of 3 or more	24 hours	43.5 %
Daily depression	Score drop of 3 or more	Over two consecutive days	36.4 %
Daily depression	Persistent score of 2 or less	Over three consecutive days	22.7 %
**Sleep**			
Variation in sleep onset	Greater than 2 hours	Over two consecutive days	36.4 %
Variation in sleep offset	Greater than 2 hours	Over two consecutive days	26.1 %
Sleep duration	More than 9 hours or less than 5 hours of sleep	One night	17.4 %
Sleep quality	Score drop of 3 or more	Over two consecutive days	36.4 %
Awakenings	3 or more per night	One night	27.3 %
Low sleep quality and late sleep period	Sleep quality score 2 or less and<br>Sleep onset after midnight with sleep offset after 9 AM	Over two consecutive days	18.2 %
**Medication**			
Medication compliance	Entered “not taking medication”	2 days or more	4.5 %
Physical activity	No registrations	2 days or more	9.1 %
Data entry into the MDB	No registrations	2 days or more	31.8 %
Other	If in doubt of participants’ condition, based on text messages or phone calls	Daily	13.0 %
