# Supplementary material for: Circadian Reinforcement Therapy in Combination With Electronic Self-Monitoring to Facilitate a Safe Postdischarge Period for Patients With Major Depression: Randomized Controlled Trial
Source: JMIR Ment Health. 2023 Nov 27;10:e50072. doi: 10.2196/50072 (PMC10714270; doi:10.2196/50072)
Supplement: Multimedia Appendix 2 [file mental_v10i1e50072_app2.pdf]

**General questions**	**Group**	**Positive**	**neutral**	**Negative**	**Inconclusive**
How have you been since starting the project?	CRT	68.4%	18.4%	13.2%	-
	TAU	53.3%	22.2%	15.6%	8.9%
What has it been like to participate in the project?	CRT	94.7%	2.6%	2.6%	-
	TAU	81.4%	11.6%	4.7%	2.3%
How as it been to manage the self-monitoring in the Monsenso Daybuilder system?	CRT	61.5%	28.2%	10.3%	-
	TAU	72.7%	15.9%	11.4%	-
How has it been to wear the Fitbit bracelet?	CRT	61.5%	28.2%	10.3%	-
	TAU	63.6%	25.0%	11.4%	-
How has it been to be phoned by investigators at the appointed timepoints and has it resulted in any changes?	CRT	92.1%	7.9%	-	-
	TAU	79.6%	18.2%	2.3%	-
What has your participation in the project meant for handling your depression after discharge?	CRT	92.5%[a]	7.5%	-	-
	TAU	62.5%	35.0%	2.5%	-
Thinking ahead, would you recommend the Monsenso Daybuilder system to others in a similar situation?	CRT	87.2%	5.1%	7.7%	-
	TAU	90.9%	-	9.1%	-
**CRT group specific questions**	**CRT**	**Positive**	**neutral**	**Negative**	**Inconclusive**
How has it been to receive psychoeducation on sleep, circadian rhythms, and depression?		76.9%	18.0%	5.1%	-
What has it meant to you to focus on your sleep in the period after discharge?		89.5%	10.5%	-	-
In what way has the psychoeducation, action plan, and Chrono diary been useful in your day-to-day life?		82.1%	18.0	-	-
Have you been phoned besides the appointed timepoints? If so, what was the outcome?		30.8%	69.2%	-	-
How was it to be phoned on these occasions?		76.0%	16.0%	8.0%	-
What has it meant to you, in general, to be part of the project and participate in the CRT group in relation to managing your depression after discharge?		92.3%	7.7%	-	-
[a] *P*<.001					
